# Individual and community level determinants of iron intake among children 6–59 months old in Ethiopia: multilevel logistic regression analysis

**DOI:** 10.1186/s12887-022-03717-0

**Published:** 2022-11-15

**Authors:** Daniel Gashaneh Belay, Melaku Hunie Asratie, Anteneh Ayelign Kibret, Kegnie Shitu, Dawit Tefera Fentie, Yalelet Fentaw Shiferaw, Baye Tsegaye Amlak

**Affiliations:** 1 Department of Human Anatomy, College of Medicine and Health Sciences, University of Gondar, Gondar, Ethiopia; 2 Department of Epidemiology and Biostatistics, Institute of Public Health, College of Medicine and Health Sciences, University of Gondar, Gondar, Ethiopia; 3 Department of Women’s and Family Health, School of Midwifery, College of Medicine and Health Sciences, University of Gondar, Gondar, Ethiopia; 4 Department of Health Education and Behavioral Sciences, Institute of Public Health, College of Medicine and Health Sciences, University of Gondar, Gondar, Ethiopia; 5 Department of Nutritional Care and Counseling, University of Gondar Specialized Hospital, Gondar, Ethiopia; 6 Department of Nursing, College of Medicine and Health Sciences, Debre Markos University, Debre Markos, Ethiopia

**Keywords:** Iron intake, Preschool, Children, And Ethiopia

## Abstract

**Background:**

Iron deficiency is one of the most important factors of anemia which is caused by poor iron intake. In addition, children need more iron because of their rapid growth. On the other side, daily intake of iron is also recommended as a standard approach for the treatment and prevention of iron deficiency anemia. In Ethiopia, although more than half of children 6–59 months of age were affected by anemia, the magnitude and factors associated with iron intake among them are understudied. Therefore this study aimed to assess the magnitude and community and individual level determinants of iron intake among 6–59 months children in Ethiopia.

**Methods:**

Demographic and Health Survey datasets (EDHS) were used for this study. The data were weighted using sampling weight to get valid statistical estimates. The total weighted samples of 9,218 children aged 6–59 months were included. A multilevel binary logistic regression model was fitted to identify factors associated with iron intake among 6–59 months of children in Ethiopia**.** In the final model adjusted odds ratio with a 95% confidence interval and *p*-value < 0.05 was taken to declare statistical significance.

**Results:**

The magnitude of iron intake among children 6–59 months in Ethiopia was 9.24% (95% CI: 8.31%, 10.15%). Individual level variables such as having at least one antenatal care visit (ANC) [AOR = 1.27; 95%CI; 1.01, 1.61], having health institution delivery [AOR = 1.46; 95%CI;1.04, 2.04], age of children ≥ 24 months [AOR = 1.82; 95%CI; 1.29, 2.57], being female child [AOR = 0.81; 95%CI; 0.67, 0.99], being greater than three birth order [AOR = 0.73, 95%CI: 0.55, 0.98], whereas community level variables such as living in large central regions [AOR = 3.68; 95%CI; 1.47, 9.21], and living in community with high women education [AOR = 1.96; 95%CI; 1.28, 2.98] have an association with iron supplements among children 6–59 months years old in Ethiopia.

**Conclusion and recommendations:**

The magnitude of iron intake among children 6–59 months old in Ethiopia is relatively low. Individual level factors such as; ANC visit, institution delivery, age of children, sex of the child, and birth order as well as; community level variables such as regions, and community women's education have a significant association with iron intake among children 6–59 months in Ethiopia. Prior attention should be given for under two years old children, children greater than three birth orders, and children living in small peripheral regions. Moreover, policymakers and other stakeholders had better plan and implement programs that empower women, enhance ANC visits, and health institution delivery to have a sustainable increment in iron intake for children in Ethiopia.

## Background

Anemia is a global public health problem that affects preschool-aged children disproportionately [1, 2]. Globally, 47.4% of preschool-aged children and in Africa, two out of three children (67.6%) are affected by anemia [2–4]. Moreover, in Ethiopia, 57% of children 6–59 months of age were touched by it [5].

Iron deficiency (ID) is one of the most important factors of anemia [2] and is caused by a lack of iron in the diet or a parasite infection in low-income countries [6, 7]. The risks for anemia in children start during gestation [6]. Low birth weight and preterm infants are born with lower iron stores and will go at increased risk of IDA [8]. The pace at which infant iron is used after delivery is determined by the infant's rate of growth, iron intake, and iron losses [7]. Because they grow so quickly, infants have higher iron demands than other age groups [9]. Iron is needed by infants in the first few months after birth to form red blood cells [7]. Furthermore, they frequently use iron stored during the latter months of pregnancy, which can lead to low or exhausted stores by the time the infant is 4–6 months old [10]. Beyond six months of age the iron content of milk is not sufficient to meet many infants requirements [9]. Prolonged milk feeding is also associated with several micronutrient deficiencies [6].

Global efforts to reduce anemia are directed toward increasing the intake of iron through intake since iron deficiency makes a large contribution [2]. Daily intake of iron is one of the World Health Organization (WHO) recommendations and is considered a standard approach for the treatment and prevention of IDA [11–13]. WHO recommended that all preschool aged children receive a course of daily iron intake where the baseline prevalence of anemia is > 40% or severe [14]. Where the diet does not include fortified foods or the prevalence of anemia in children at approximately one year of age is severe (above 40%), supplements of iron at a dosage of 2 mg/kg of body weight per day should be given to all children [9].

The implementation of iron intake for children was reported by 40% of countries [4]. The weekly iron and folic acid intake program in a province of Viet Nam resulted in a 48% reduction in the prevalence of anemia [4]. A study conducted in India showed that there is a significant negative correlation between iron utilization and anemia among children [13]. Moreover, the study showed that low iron intake causes decreased physical capacity and reduced immunity, low cognitive development, and retarded growth in preschool children [15]. But in Ethiopia, the magnitude of iron intake among those under five at the national level is understudied. Therefore this study aimed to assess the magnitude and community and individual level determinants of iron intake among 6–59 months children in Ethiopia.

## Methods

### Study design, setting, and period

This study was based on the recent standard Ethiopian Demographic and Health Survey (EDHS, 2016) data, which was crossectional and conducted from January 18, 2016, to June 27, 2016. The standard EDHS data set is with large sample size and is used to get all parameters that can be representative of the source [16].

Ethiopia is an East African country found in 3^0^ -14^0^ North and 33^0^—48^0^Eastern directions with 1.1 million Sq.km coverage. It is the second-most populous country in Africa with an estimated population of 114,963,588 in 2021 [17]. Administratively, Ethiopia is federally decentralized into two city administrations and nine regions. Kebele is the lowest administrative unit [16].

### Populations

#### Source and study population

The source population was all children aged 6–59 months preceding five years of the survey period in Ethiopia whereas, children aged 6–59 months preceding five years of the survey period in the selected Enumeration Areas (EAs) were the study population. Children who have incomplete data for the outcome variable were excluded from the analysis.

### Sample size determination and Sampling method

The most recent standard census of Ethiopia (2016 EDHS) was used. DHS samples are stratified by geographic region and by urban/rural areas within each region. DHS sample designs are usually two-stage probability samples drawn from an existing sample frame. Enumeration Areas (EAs) were the sampling units for the first stage of sampling. In selected EAs, households (HHs) comprise the second stage of sampling. Following the listing of the households, a fixed number of households is selected by equal probability systematic sampling in the selected cluster [16]. The detailed sampling procedure was available in each DHS report from the Measure DHS website [16].

To restore the representativeness of the sample data, weighted values were used and calculated from children’s records or kid’s records (KR) datasets. Finally, a total weighted sample of 9,218 children in the age category of 6–59 months was included in this study.

### Study variables

The outcome variable of this study was iron intake in children 6–59 months. During the survey, their mother was asked questions about their living children 6–59 months who received iron pills, sprinkles, or syrup in the seven days preceding the interview [16].

Individual and community-level independent variables have been studied. The individual-level factors include socio-demographic characteristics such as; the age of the mother, marital status, mother's employment, maternal education, family size, household wealth status, and media exposure were included. Child-related factors such as the sex of the child, age of the child, the plurality of birth, birth order, and breastfeeding status are all considered. Health service utilization-related factors such as pregnancy wontedness, place of delivery, PNC, and ANC visit were also considered. The community-level factors include; distance from health facilities, place of residence, region, community level media exposure, community level women's education, and community poverty were considered.

Community-level poverty was determined by the proportion of women in the poor and poorest quintile. It was coded as “0” for low (communities in which < 50% women had poor and poorest wealth quintiles), “1” for high (communities in which ≥ 50% women had poorest and poorer wealth quintiles) poverty communities. Community-level media exposure was assessed by the proportion of women who had at least been exposed to one media. It employs "0" to indicate low-level and "1" to indicate high-level media coverage at the community level, the same method is also used for community level women's education [18, 19].

### Data processing and analysis

Data from the dataset were downloaded as standard DHS data in STATA format and then cleaned, integrate, transformed, and append to produce favorable variables for the analysis. Microsoft Excel was used to generate descriptive analysis and for aggregation of some community level variables whereas STATA 14 software was used for analytic statistics.

### Model building for multi-level analysis

The DHS data has hierarchical nature, and children aged 6–59 months were nested within a cluster. This might violate the independence and equal variance assumptions. Therefore, a multilevel binary logistic regression model was fitted. Four models were fitted for multi-level analysis. The first was the null model (Model 1) containing no exposure variables which was used to check the variability across the cluster. The second (Model 2) and the third (Model 3) multilevel models contain individual-level variables and community-level variables respectively. In the fourth model (Model 4), both individual and community level variables were fitted simultaneously with the prevalence of iron intake among those under five.

$$Log \left(\frac{\pi ij}{1-\pi ij}\right)=\beta o+ \beta 1xij+ \beta 2xij+\dots uj+eij$$

where,$\pi ij$: the probability of iron intake, $1-\pi ij$: the probability of no iron intake. While the ß0 is the intercept that is the effect on feeding iron intake when the effect of all explanatory variables is absent. $\beta 1$ xij are individual and community level variables for the ith individual in group j, respectively. Moreover, the ß’s are fixed coefficients indicating a unit increase in *X* can cause a *ß* unit increase in the probability of iron intake. The UK shows the random effect (effect of the community on the mother’s decision to provide iron intake) for the j^th^ community. The clustered data nature and the within and between community variations were taken into account assuming each community has a different intercept (β0) and fixed coefficient (β) [18, 20, 21].

Model comparisons were done using the likelihood test and the model with the highest value was model 4 and selected as the best-fitted model. The variance inflation factor (VIF) was used to detect multicollinearity, and all variables had VIF values less than 10 and the mean VIF value of the final model was 1.50.

The association between dependent and independent variables was assessed using Crude Odds Ratio (COR) and the Adjusted Odds Ratio (AOR). Factors with a *p*-value ≤ 0.2 in COR have been selected as candidates for the final model. Associations between dependent and independent variables were assessed and their strength has been presented using adjusted odds ratios and 95% confidence intervals with a *p*-value of < 0.05.

The measure of variation was estimated using the following parameters; the Median Odds Ratio (MOR) is defined as the median value of the odds ratio between the area at the lowest risk and the highest risk when randomly picking out two clusters. ${{\mathrm{MOR}=e}^{0.95}}^{\sqrt{VA}}$ or, MOR = exp.[√(2 × VA) × 0.6745], where; VA is the area level variance [18, 20, 22]. The Proportional Change in Variance (PCV reveals the variation in bottle-feeding among children 0–23 months explained by factors. The PCV is calculated as; $PCV=\frac{Vnull-VA}{V null}*100\%$ where; Vnull = variance of the initial model, and VA = variance of the model with more terms. Moreover, Intra Class Correlation Coefficient (ICC), which reveals the variation of bottle feeding between clusters is calculated as;$ICC=\frac{VA}{VA+3.29}*100\%$, where; VA = area/cluster level variance [18, 20, 22].

## Results

### Socio demographic characteristics of mothers or caregivers

In this study, a total weighted sample of 9,218 children of age 6–59 months were included. More than half (53.81%) of mothers of children were in the age group of 20–35 years, with a median age of 29 (IQR: 25, 34) years. Three-fifths of women (66.75%) had no formal education. Most (88.92%) of the respondents were rural inhabitants. Ninety percent (90.15%) of women included in the study were from large central regions (Table 1).

**Table 1:** Socio-demographic characteristics of the study mothers/caregivers in study determinants of iron supplements among children 6–59 months in Ethiopia: based on 2016 EDHS

Variables	Categories	Weighted Frequency (n)	Weighted Percentage (%)
Age of women (years)	15–19	1,920	20.83
	20–34	4,960	53.81
	35–49	2,338	25.36
Sex of household head	Male	7,926	85.99
	Female	1,291	14.01
Educational attainment of women	No education	67	66.75
	Primary education	2,434	26.41
	Secondary & above	631	6.84
Occupation of women	Not working	5,047	54.76
	Worked	4,170	45.24
Marital status of the mother	Married	8,638	93.71
	Not married	580	6.29
Household family size	1–4	2,403	26.07
	5–10	6,517	70.7
	> 11	297	3.23
Media exposure	No	6,217	67.45
	Yes	3,000	32.55
Wealth index	Poorest	4,313	46.79
	Middle	1,939	21.04
	Richest	2,966	32.17
Community level women's education	Low	4,731	51.33
	High	4,486	48.67
Community level media usage	Low	4,072	44.17
	High	5,146	55.83
Community level poverty	Low	5,637	61.16
	High	3,579	38.84
Residence	Urban	1,021	11.08
	Rural	8,196	88.92
Region	Metropolis	270	2.93
	Large central	8,310	90.15
	Small periphery	638	6.92

### Child related characteristics and health service utilization-related factors

From the total weighted sample of 9,218 children of age 6–59 nearly similar proportions of males (51.63%) and females (48.37%) were studied. Nearly three-fifths (66.64%) of the children were above two years with a median age of 32 (IQR: 18 46,) months. 66.35% of the child were singleton. Moreover, three fourth (74.82%) of infants were delivered at home (Table 2).

**Table 2:** Child related characteristics and health service utilization-related factors of iron supplements among children 6–59 months old in Ethiopia: based on 2016 EDHS

Variables	Categories	Weighted Frequency(n)	Weighted Percentage (%)
Sex of child	Male	4,759	51.63
	Female	4,458	48.37
Age of child	6–11 months	1,071	11.62
	12–23 months	2,004	21.74
	> 23 months	6,142	66.64
Plurality	Single	9,006	97.7
	Multiple	212	2.3
Birth order	≤ 3	4,508	48.9
	> 3	4,710	51.1
Breastfeeding status	No	3,102	33.65
	Yes	6,116	66.35
Pregnancy wontedness	Wanted	6,559	71.16
	Unwanted	2,658	28.84
ANC visits	No ANC	2,258	37.03
	At least one ANC	3,839	62.97
Place of delivery	Home delivery	6,897	74.82
	Health facilities	2,321	25.18
Distance from health facilities	Not big problem	3,622	**39.29**
	**Big problem**	**5,596**	**60.71**

### The magnitude and associated factors of iron intake among children 6–59 months old in Ethiopia

The magnitude of iron intake among children 6–59 months old in Ethiopia was 9.24% (95% CI: 8.31, 10.15).

In the random-effects analysis, the null model ICC showed that 34.4% of the variations in iron supplements among children 6–59 months old were attributed to cluster differences. The median odds ratio (MOR) between the lower-risk and higher-risk area of iron supplement among clusters were 3.34. Moreover, the final model (model four) expressed about 21.89% of the variation in iron supplements among 6–59 months old children. Log-likelihood and deviance were used for model comparison and model four has the lowest deviance (3386) and the highest log likelihood (-1693) which is considered the best fit model (Table 3).

**Table 3:** Multilevel (individual and community level) analysis of factors associated with iron intakes among children 6–59 months old in Ethiopia, EDHS 2016

Variables	Categories	^Null model^	^a^Model 2 AOR [95% CI]	Model 3 AOR [95% CI]	Model 4 AOR [95% CI]
Age of women (years)	15–24	_**––**_	1.00	_**––––––**_	1.00
	25–34	_**––**_	1.33 [0.99, 1.79]	_**––––––**_	1.34 [0.99, 1.79]
	36–49	_**––**_	1.13 [0.76, 1.67]	_**––––––**_	1.13 [0.77, 1.67]
Sex of household head	Male	_**––**_	1.00	_**––––––**_	1.00
	Female	_**––**_	0.67 [0.48, 0.95]*	_**––––––**_	0.73 [0.48, 1.05]
Educational attainment of women	No education	_**––**_	1.00	_**––––––**_	1.00
	Primary education	_**––**_	1.11 [0.86, 1.41]	_**––––––**_	1.03 [0.80, 1.31]
	Sec.& above	_**––**_	1.49 [0.97, 2.29]	_**––––––**_	1.37 [0.89, 2.11]
Occupation of women	Not worked	_**––**_	1.00	_**––––––**_	1.00
	Worked	_**––**_	0.933 [0.76, 1.15]	_**––––––**_	0.94 [0.76, 1.15]
Marital status of the mother	Married	_**––**_	1.00	_**––––––**_	
	Not married	_**––**_	0.83 [0.53, 1.31]	_**––––––**_	0.84 [0.53, 1.32]
Household family size	1–4	_**––**_	1.00	_**––––––**_	1.00
	5–10	_**––**_	1.46 [1.12, 1.91]*	_**––––––**_	1.45 [1.11, 1.89]**
	> 11	_**––**_	1.66 [0.88, 3.14]	_**––––––**_	1.69 [0.89, 3.19]
Media exposure	No	_**––**_	1.00	_**––––––**_	1.00
	Yes	_**––**_	1.03 [0.8, 1.31]	_**––––––**_	1.02 [0.79, 1.30]
Wealth index	Poorest	_**––**_	1.00	_**––––––**_	1.00
	Middle	_**––**_	1.22 [0.93, 1.61]	_**––––––**_	1.17 [0.88, 1.55]
	Richest	_**––**_	1.37 [1.03, 1.82]*	_**––––––**_	1.29 [0.96, 1.73]
Sex of child	Male	_**––**_	1.00	_**––––––**_	1.00
	Female	_**––**_	0.64 [0.46, 0.90]*	_**––––––**_	0.81 [0.67, 0.99]**
Age of child	6–11	_**––**_	1.00	_**––––––**_	1.00
	12–23	_**––**_	1.47 [1.07, 2.03]*	_**–––––-**_	1.49 [1.08, 2.06]**
	> 24	_**––**_	1.86 [1.32, 2.63]***	_**–––––-**_	1.82 [1.29, 2.57]**
Plurality of birth	Single	_**––**_	1.00	_**––––––**_	1.00
	Multiple	_**––**_	0.84 [0.34, 2.08]	_**––––––**_	0.82 [0.33, 2.02]
Birth Order	< 3	_**––**_	1.00		1.00
	> 3	_**––**_	0.74 [0.55, 0.98]**		0.73 [0.55, 0.98]*
Breastfeeding status	No	_**––**_	1.00		1.00
	Yes	_**––**_	0.86 [0.67, 1.11]		0.87 [0.678, 1.11]
Pregnancy wontedness	Wanted	_**––**_	1.00	_**––––––**_	1.00
	Unwanted	_**––**_	0.82 [0.65, 1.03]	_**––––––**_	0.81 [0.64, 1.02]
ANC visits	No ANC	_**––**_	1.00	_**––––––**_	1.00
	At least one ANC	_**––**_	1.29 [1.03, 1.64]*	_**––––––**_	1.27 [1.01, 1.61]**
Place of delivery	Home delivery	_**––**_	1.00	_**––––––**_	1.00
	Health facilities	_**––**_	1.46 [1.04, 2.04]*	_**––––––**_	1.46 [1.04, 2.04]*
**Community level variables**					
Distance from health facilities	Not big problem	_**––**_	_**––––––**_	1.00	1.00
	Big problem	_**––**_	_**––––––**_	1.16 [0.96, 1.41]	1.09 [0.87, 1.39]
Residence	Urban	_**––**_	_**––––––**_		1.00
	Rural	_**––**_	_**––––––**_	1.09 [0.66, 1.81]	1.54 [0.84, 2.84]
Region	Metropolis	_**––**_	_**––––––**_	1.00	1.00
	Large central	_**––**_	_**––––––**_	3.39 [1.53, 7.52]*	3.68 [1.47, 9.21]**
	Small periphery	_**––**_	_**––––––**_	1.74 [0.70, 4.31]	2.56 [0.88, 7.39]
Community-women education	Low	_**–-**_	_**––––––**_	1.00	1.00
	High	_**––**_	_**––––––**_	2.13 [1.48, 3.06]**	1.96 [1.28, 2.98]**
Community media usage	Low	_**–-**_	_**––––––**_	1.00	1.00
	High	_**––**_	_**––––––**_	1.02 [0.70, 1.48]	0.92 [0.59, 1.42]
Community poverty	Low	_**––**_	_**––––––**_	1.00	1.00
	High	_**––**_	_**––––––**_	0.81 [0.56, 1.19]	0.82 [0.52, 1.30
**Random effect**					
	Community level variance	1.69	1.54	1.58	1.32
	ICC	34.4%	32.35%	32.92%	29.73%
	MOR	3.44			
	PCV	Ref	8.87%	6.51%	21.89%
**Model comparison**					
	Logliklihood	-2611	-1707	-2588	-1693
	Deviance	5222	3414	5176	3386

* = *P*-value < 0.05

** = *P* value < 0.01

*** = *P* value < 0.001

*ICC* Inter cluster correlation coefficient, *MOR* Median odds ratio, *PCV* Proportional change in variance, *AOR* Adjusted odds ratio, *CI* Confidence interval, *Ref* Reference

In the fixed effect analysis, individual level factors such as; ANC visit, institution delivery, age of children, sex of the child, family size, and birth order have a statistically significant association with iron intake among children 6–59 months old.

In this study, as the age of children increased to 12–23 months old, and > 24 months old, they were 1.49 and 1.82 times more likely to get iron supplements as compared to 6–11 months old children [AOR = 1.49; 95%CI; 1.08, 2.06] and [AOR = 1.82; 95%CI; 1.29, 2.57].

In our study, mothers who had at least one ANC visit during pregnancy, and had health institution delivery had 1.27 and 1.46 times more likely to access their child's iron supplements as compared to no ANC visit and home delivery mothers [AOR = 1.27; 95%CI;1.01, 1.61], and [AOR = 1.46; 95%CI;1.04, 2.04] respectively.

In our study, female children were 19% less likely to have iron supplements as compared to males [AOR = 0.81; 95%CI; 0.67, 0.99]. The odds of getting iron intake among children who have a birth order greater than three were decreased by 27% [AOR = 0.73, 95%CI: 0.55, 0.98].

Moreover, community level factors such as; regions and community level women's education have a statistically significant association with iron intake among children 6–59 months old.

In this study, those living in a large central region were 3.68 times more likely to have iron intake [AOR = 3.68; 95%CI; 1.47, 9.21]. Women who were living in high community level education were 1.28 times more likely to have iron supplements among children 6–59 months in Ethiopia [AOR = 1.96; 95%CI; 1.28, 2.98] (Table 3).

## Discussions

In Ethiopia, 57% of children 6–59 months of age were affected by anemia [5]. World Health Organization (WHO) recommended that all preschool-aged children receive a course of daily iron intake where the baseline prevalence of anemia is severe (> 40%) [14]. Therefore this study aimed to determine the magnitude and associated factors of iron supplements among 6–59 months children in Ethiopia. Based on this, the magnitude of iron intake among children 6–59 months old in Ethiopia was 9.24% (95% CI: 8.31, 10.15). This is higher than in Afghanistan (6%) [6] but lower than in a study conducted in India (24.1%) [15]. This might be due to the implementation difference in nutritional programs including iron supplements and the socioeconomic difference between countries [15].

In this study, as the age of children increases, they are more likely to get iron. This is supported by a study in India [15]. A study in Ethiopia also showed that as a child ages increase the probability of being anemic decreases [23]. This is common because most mothers think that, the child can resist medication side effect when the age becomes increase and most supplements and deworming have been given in Ethiopia during preschool ages.

In our study, mothers who had at least one ANC visit during pregnancy, and had health institution delivery have more likely to access their child's iron supplements as compared to no ANC visit and home delivery mothers. This is because of having information and knowledge during follow up, mothers who have ANC follow have a positive health behavior like utilization of iron for them and their child as well to prevent anemia [23].

In this study, female children were less likely to have iron supplements than males. This is might be due to the caregiving behaviors of mothers because of preference. In Ethiopia, parents give more meal freedom and caretaking to male children than to females [24]. In this study higher birth order has a negative association with iron supplements among children 6–59 months. This is in line with a study in India [15], and a study in Asia and India [25] which show when birth order increases the use of iron supplements decreases. A study conducted in Ethiopia among 6–59 months children showed that higher birth-order children had higher odds of anemia than first-order children [23]. This is because birth order matters in the care taken and the nutritional status of children [26].

Living in high women's education community has a positive association with iron supplements among children 6–59 months in Ethiopia. This is supported by a study in Afghanistan [6] and a study in India [15]. This might be due to the that, educated mothers’ had health advantages in understanding the information [24]. They ensure better dietary practices for children, including iron-rich food consumption, and taking iron intake [13].

In our study children who were living in large central regions were more likely to have iron supplements than metropolitan ones. This is supported by a study that showed that Children from Dire Dawa, Harrie, Afar, and Somali (metropolis and small peripheral regions) had higher odds of anemia than children from the Tigray region (large central) [23]. This might due to that large central regions has increased accessibility and affordability of maternal and child health service when compared to small peripheral which is an emerging region [27].

The main strength of this study was the use of the weighted nationally representative data with a large sample which makes it representative at country levels. Therefore, it has appropriate statistical power that can be generalized of the estimates in iron supplements in the study setting to all children 5–59 months during the study period. Since the data were collected cross-sectional by self-reported interviews would be prone to recall and social desirability bias. The drawback of the secondary nature of data was inevitable.

## Conclusion

The magnitude of iron intake among children 6–59 months old in Ethiopia is relatively low. Individual level factors such as; ANC visit, institution delivery, age of children, sex of the child, and birth order as well as; community level variables such as regions, and community women's education have a significant association with iron intake among children 6–59 months in Ethiopia. Prior attention should be given for under two years old children, children greater than three birth orders, and children living in small peripheral regions. Moreover, policymakers and other stakeholders had better plan and implement programs that empower women, enhance ANC visits, and health institution delivery to have a sustainable increment in iron intake for children in Ethiopia.

## Data Availability

Data is available online from the “measures DHS program” and taken after writing a concept note to the DHS program and getting permission to use it. Then register through this website, https://dhsprogram.com/data/new-user-registration.cfm.

## References

[1] World Health Organization: Prevalence of anaemia in children aged 6–59 months. Available in: https://www.who.int/data/gho/indicator-metadata-registry/imr-details/4801. Accessed 08 June 2022.

[2] McLean E (2009). Worldwide prevalence of anaemia, WHO vitamin and mineral nutrition information system, 1993–2005. Public Health Nutr.

[3] Stevens GA (2013). Global, regional, and national trends in haemoglobin concentration and prevalence of total and severe anaemia in children and pregnant and non-pregnant women for 1995–2011: a systematic analysis of population-representative data. The Lancet Global Health.

[4] World Health Organization: Global nutrition policy review: What does it take to scale up nutrition action? Available in: https://www.knowledge-action-portal.com/en/content/global-nutrition-policy-review-what-does-it-take-scale-nutrition-action. Accessed 08 June 2022.

[5] Central Statistical Agency Addis Ababa, Ethiopia. Ethiopia Demographic and Health Survey 2016. The DHS Program ICF Rockville, Maryland, USA. 2017.

[6] Barekzai A, Baraki B (2021). Iron supplementation among children aged 6 to 59 months in Afghanistan: a report of Afghanistan Demographic and Health Survey (AFDHS) 2015. J Nutr Food Sci..

[7] Domellöf M (2014). Iron requirements of infants and toddlers. J Pediatr Gastroenterol Nutr..

[8] Long H (2012). Benefits of iron supplementation for low birth weight infants: a systematic review. BMC Pediatr.

[9] Organization W.H. (2013). Essential nutrition actions: improving maternal, newborn, infant and young child health and nutrition.

[10] Chaparro CM (2008). Setting the stage for child health and development: prevention of iron deficiency in early infancy. The Journal of nutrition.

[11] Thompson J, Biggs B-A, Pasricha S-R (2013). Effects of daily iron supplementation in 2-to 5-year-old children: systematic review and meta-analysis. Pediatrics.

[12] WHO. Guideline: Intermittent iron supplementation in preschool and school-age children. Geneva: World Health Organization; 2011. 24479203

[13] Thomas MS, Demirchyan A, Khachadourian V (2020). How Effective is iron supplementation during pregnancy and childhood in reducing anemia among 6–59 months old children in India?. Front Public Health.

[14] Stoltzfus RJ, Dreyfuss ML. Guidelines for the use of iron supplements to prevent and treat iron deficiency anemia. Washington, DC: Ilsi Press; 1998.

[15] Srivastava S, Kumar S (2021). Does socio-economic inequality exist in micro-nutrients supplementation among children aged 6–59 months in India? evidence from national family health survey 2005–06 and 2015–16. BMC Public Health.

[16] Croft TN, Aileen M, Marshall J, Allen CK, et al. Guide to DHS Statistics. Rockville: ICF; 2018.

[17] Worldometer: African Countries by Population in 2021 - Available in: https://www.worldometers.info/worldpopulation/africa-population/. Accessed 08 June 2022.

[18] Collin M. "Lining up to eat: Birth order and nutritional status in rural Ethiopia." Oxford: St. Antony’s College; 2006.

[19] Teshale AB, Tesema GA (2020). Prevalence and associated factors of delayed first antenatal care booking among reproductive age women in Ethiopia; a multilevel analysis of EDHS 2016 data. PloS one.

[20] Merlo J (2005). A brief conceptual tutorial on multilevel analysis in social epidemiology: interpreting neighbourhood differences and the effect of neighbourhood characteristics on individual health. J Epidemiol Community Health.

[21] Tessema ZT (2021). Pooled prevalence and determinants of modern contraceptive utilization in East Africa: A Multi-country Analysis of recent Demographic and Health Surveys. PloS one.

[22] Merlo J (2005). A brief conceptual tutorial of multilevel analysis in social epidemiology: linking the statistical concept of clustering to the idea of contextual phenomenon. J Epidemiol Community Health.

[23] Gebremeskel MG (2020). Individual and community level factors associated with anemia among children 6–59 months of age in Ethiopia: a further analysis of 2016 Ethiopia demographic and health survey. PLoS One.

[24] Abeway S (2018). Stunting and its determinants among children aged 6–59 months in northern Ethiopia: a cross-sectional study. J Nutr Metab.

[25] Kotecha PV (2011). Nutritional anemia in young children with focus on Asia and India. Indian J Community Med.

[26] Collin, M., Lining up to eat: Birth order and nutritional status in rural Ethiopia. St. Antony’s College, Oxford, 2006

[27] Teshale AB, Tesema GA (2020). Magnitude and associated factors of unintended pregnancy in Ethiopia: a multilevel analysis using 2016 EDHS data. BMC Pregnancy and Childbirth.

